# *CCR7* and its related molecules may be potential biomarkers of pulmonary arterial hypertension

**DOI:** 10.1002/2211-5463.13130

**Published:** 2021-05-12

**Authors:** Mengsi Cai, Xiuchun Li, Haoru Dong, Ying Wang, Xiaoying Huang

**Affiliations:** ^1^ Key Laboratory of Heart and Lung Department of Respiratory and Critical Care Medicine The First Affiliated Hospital of Wenzhou Medical University Zhejiang China

**Keywords:** bioinformatics, competitive endogenous RNA mechanism, long noncoding RNA, microRNA, pulmonary arterial hypertension

## Abstract

Pulmonary arterial hypertension (PAH) is a chronic progressive cardiovascular disease characterized by vascular remodeling and leading to right‐heart failure. The purpose of this research was to further study the pathogenesis of PAH and to detect potential prognostic signatures. Differentially expressed genes (DEGs) selected from GSE38267 were mostly enriched in inflammation‐related pathways, suggesting inflammation may be involved in the occurrence and development of PAH. Through the prediction and verification of related miRNAs and long noncoding RNAs using online databases and Gene Expression Omnibus (GEO) datasets, *CCR7* and its related molecules, including *hsa‐let‐7e‐5p* and *SNHG12*, were identified as possible targets. The expression levels of *CCR7*, *hsa‐let‐7e‐5p* and *SNHG12* were then verified by quantitative RT‐PCR *in vivo* and *in vitro*. Further study showed that silencing of *SNHG12* decreased the expression of *CCR7* under hypoxia treatment *in vitro*. Dual‐luciferase reporter assays were used to verify the relationship between *hsa‐let‐7e‐5p* and *SNHG12*. Collectively, our research reveals that a long noncoding RNA–miRNA–mRNA interaction network may be involved in the pathogenesis of PAH and suggests *SNHG12*, *hsa‐let‐7e‐5p* and *CCR7* as potential biomarkers for PAH.

## Graphical abstract

Pulmonary arterial hypertension (PAH) is a chronic progressive cardiovascular disease characterized by vascular remodeling, leading to right‐heart failure. In this study, we detected the pathways and potential molecules related to PAH through bioinformatics and experiments *in vivo* and *in vitro*. We identified *SNHG12*, *hsa‐let‐7e‐5p* and *CCR7* as potential biomarkers for PAH.

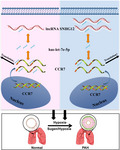

**Abbreviations**
ceRNA: competing endogenous RNA
CIH: chronic intermittent hypoxia
DEG: differentially expressed gene
EC: endothelial cell
FC: fold change
GEO: Gene Expression Omnibus
GSEA: gene set enrichment analysis
GO: Gene Ontology
HC: healthy control
hPASMC: human pulmonary artery smooth muscle cell
KEGG: Kyoto Encyclopedia of Genes and Genomes
lncRNA: long noncoding RNA
LV + S: left ventricle plus the interventricular septum
MUT: mutation
ncRNA: noncoding RNA
NF‐κB: nuclear factor‐κB
Nx: normoxia
PAH: pulmonary arterial hypertension
qRT‐PCR: quantitative RT‐PCR
RV: right ventricle
RVSP: right ventricular systolic pressure
Su/Hx: Sugen 5416–hypoxia
WT: wild‐type

Pulmonary arterial hypertension (PAH) is a kind of pulmonary circulation disease characterized by increased pulmonary vascular resistance and rising pulmonary artery pressure [1, 2]. For the past several decades, PAH has continued to be a disease connected to a high morbidity and mortality whose morbidity has been estimated to be 15 cases per million in spite of enlightened treatment [3]. Notwithstanding many biological processes and biomolecules, such as oncogenes and tumor suppressors, having been reported to be involved in the development of PAH [4], the pathogenesis of PAH and related biomarkers still needs to be researched. Therefore, there is an urgent need to detect the potential mechanism of PAH and find related biomarkers.

Noncoding RNA (ncRNA) is a type of RNA that is not translated into proteins but still contains information and performs vital functions [5]. It has been discovered to act not only as an important regulator of diseases, including various cancers, central nervous system disorders and cardiovascular diseases [6], but also as an active participant in numerous biological processes, such as chromatin remodeling, gene expression and signal transduction [7]. Long noncoding RNA (lncRNA) is a type of ncRNA no shorter than 200 nucleotides [8], which is involved in plenty of cellular activities. Correspondingly, Sun et al. [9] pointed out that metformin could prohibit PAH from developing and lncRNA *NONRATT015587.2* could possibly be a biomarker for the diagnosis of PAH. Competing endogenous RNA (ceRNA) refers to a new gene‐regulatory mechanism that may cause mediation and alternations in key subpathway regions [10]. In previous research, Feng et al. [10] identified several ceRNA‐mediated subpathways that were relevant with PAH via a method termed as ce‐Subpathway. However, there is still insufficient evidence on the ceRNA regulatory mechanism for PAH, and these inspire our great interest.

In this study, we used bioinformatics methods to analyze and compare the raw sequencing data of the whole blood of patients with PAH and healthy people, hoping to screen out mRNA, miRNA and lncRNA, which may play key roles in PAH, so as to uncover the pathogenesis of PAH from the molecular level and explore potential biomarkers.

## Material and methods

### The identification of DEGs

The microarray dataset (GSE38267) based on platform GPL13607 was downloaded using "GEOquery" package in the r software. GSE38267 is a microarray analysis performed with homogeneous groups of patients with cystic fibrosis (*n* = 23), patients with PAH (*n* = 13) and healthy control subjects (HCs; *n* = 28). To explore PAH‐related molecules, we selected blood samples from patients with PAH and HCs. For genes represented by more than one probe set, we took the average of the expression values of these probe sets. Then the "limma" package was used for standardization. The DEGs were screened out using the "limma" package by the following selection criteria: *P* < 0.05 and fold change (FC) > 2.

### Enrichment analyses

The Gene Ontology (GO) and Kyoto Encyclopedia of Genes and Genomes (KEGG) pathway enrichment analyses of the DEGs, which are commonly used approaches for functional studies, were conducted with the “clusterprofiler” package in r software. The results were visualized by “goplot” package.

Gene set enrichment analysis (GSEA), a widely used software package, was also performed using the "clusterprofiler" package in r software. We chose “h.all.v7.1.symbols.gmt” and "c2.all.v7.1.symbols.gmt" downloaded from MSigDB (http://software.broadinstitute.org/gsea/msigdb/index.jsp) as the reference gene sets, which were read into r software using the "GSEABase" package. The top three GSEA terms were visualized by “ggplot2” package.

### Protein protein interaction network construction and gene clusters identification

A protein protein interaction (PPI) network based on the DEGs was carried out with STRING (https://string‐db.org/) and the interaction scores > 0.400. Then the result of STRING analysis was uploaded into cytoscape v3.7.1, and MCODE plug‐in was performed to screen out densely connected clusters in the PPI network. Furthermore, the genes in the top three gene clusters were separately imported into STRING to generate the PPI network and explore related biological processes.

### Prediction of related miRNAs

Three online bioinformatics prediction tools (TargetScanv7.1, miRDB, miRWalk) were used to predict the target miRNAs of the candidate genes cluster. To ensure the accuracy of the prediction, we took the intersection of three online databases as the final result. Further study was performed by cytoscape v3.7.1, and miRNAs that regulate more than two genes were selected for the following research.

### miRNAs enrichment analysis

miEAA2.0 (https://ccb‐compute2.cs.uni‐saarland.de/mieaa2) is a website that provides miRNA overrepresentation/underrepresentation analysis and GSEA for 10 species by the enrichment methods: overrepresentation analysis or GSEA. The pivotal miRNAs were imported to the miEAA2.0 tool.

### Prediction of miRNA–target lncRNA

lncRNAs regulated by the selected miRNAs were predicted in starBase (http://starbase.sysu.edu.cn/), which is a significant database for researching ncRNAs. The obtained results were comparable with the differentially expressed lncRNAs analyzed from lncRNA datasets (GSE38267).

### Preparation of the animal models

Male wild‐type (WT) C57BL/6J mice (20–25 g) were purchased from Vital River Laboratory Animal Technology (Beijing, China). All mice were housed under standard conditions (temperature, 20–24 °C; 12 : 12‐hour light : dark cycle) and fed with a standard rodent diet and water in a specific pathogen‐free animal facility. All procedures were approved by the Animal Ethics Committee of Wenzhou Medical University.

As for chronic intermittent hypoxia (CIH)‐induced pulmonary hypertension models, after adapting for 1 week, all mice were randomly assigned to two groups, with *n* = 6 per group: (a) normoxia (Nx) group and (b) CIH. The CIH group was housed in a closed hypoxia chamber (8–11% O_2_, 8 h per day, 6 days per week), while the Nx group was exposed to room air. The hypoxia exposure continued for 4 weeks.

As for the Sugen 5416–hypoxia (Su/Hx)‐induced pulmonary hypertension mouse model, after adapting for 1 week, all mice were randomly assigned to two groups, with *n* = 5 per group: (a) Nx group and (b) Su/Hx group. The mice in the Su/Hx group experienced a weekly subcutaneous injection of Sugen 5416 (20 mg·kg^−1^) and were housed in a normobaric hypoxic chamber (10% O_2_, 24 h per day, 7 days per week), while the Nx group was exposed to room air. The Su/Hx exposure continued for 3 weeks.

### Measurement of hemodynamics and right ventricular hypertrophy

After CIH‐induced pulmonary hypertension model establishment, mice were anesthetized and fixed on an operating table. The right ventricular systolic pressure (RVSP) was measured by pressure transducers (PowerLab 8/35 multichannel biological signal recording system; AD Instruments, Australia). To further evaluate the right ventricular hypertrophy, the left ventricle plus the interventricular septum (LV + S) and the right ventricle (RV) tissue were dissected and weighed separately.

### Hematoxylin and eosin staining

The right lung was separated and fixed with 4% paraformaldehyde for 48 h. Then the samples were embedded in paraffin, sectioned routinely and hematoxylin and eosin stained. The pulmonary arteries were captured randomly with a microscope (Nikon, Tokyo, Japan). The ratios of the wall thickness to the total thickness and the ratios of the pulmonary artery wall area to the total area were measured and analyzed using the ipps 6.0 image processing system.

### Cell culture and siRNA transfection

Human pulmonary artery smooth muscle cells (hPASMCs) were purchased from ATCC and were incubated in a culture bottle of Smooth Muscle Cell Medium with 2% FBS, 1% Smooth Muscle Cell Growth Supplement, 100 μg·mL^−1^ streptomycin and 100 U·mL^−1^ penicillin (all these were purchased from Gibco, Grand Island, NY, USA). The Nx group was cultured in a humidified incubator (37°C, 21% O_2_, 5% CO_2_, 74% N_2_). The hypoxia‐treated group was cultured in a humidified incubator (37 °C, 5% O_2_, 5% CO_2_, 90% N_2_) for 24 h. hPASMCs were transfected with siRNAs, which were purchased from Ribobio (Guangzhou, China) according to the manufacturer’s protocol. The efficiency of knockdown was assessed by quantitative RT‐PCR (qRT‐PCR) after 24 h.

### Quantitative RT‐PCR analysis for mRNA, miRNA and lncRNA

mRNA and lncRNA were isolated from lung tissue of mice and hPASMCs with the RNeasy mini kit (Qiagen, USA) and then reverse transcribed into cDNA using the iScript cDNA Synthesis Kit. Total miRNA was extracted from lung tissue of mice and hPASMCs using SanStep Column microRNA Extraction Kit (Sangon Biotech, Shanghai, China) and then reverse transcribed into cDNA by miRNA First Strand cDNA Synthesis (stem‐loop method) (Sangon Biotech). Amplification of cDNA was carried out by a quantitative RT‐PCR (CFX96 Real‐Time System; Bio‐Rad, Hercules, CA, USA).

The target gene mRNA and lncRNA expression were standardized with the housekeeping gene *GAPDH*, while *U6* was used as the endogenous control of miRNA. All samples were tested in triplicate. The primer sequences were shown in Table 1.

**Table 1:** Primers used for quantitative RT‐PCR in this study. F, forward; R, reverse.

Primer sets name	Reverse transcriptase primer (5′–3′)	Real‐time quantitative PCR primer (5′–3′)
*CCR7* (mouse)	/	F: GCTGAGATGCTCACTGGTCA R: AGCCAGCATAGGCACTAGGA
*CCR7* (human)	/	F: CCCACAGACTCAAATGCTCA R: CTTTTCCTCACCAAGCCAAG
*SNHG12* (mouse)	/	F: GATTTTTCCGTCTGGTCCAA R: TGGTCTCCCTCCTCACAATC
*SNHG12* (human)	/	F: AAGCCTCTGCCTGCCTTC R: GCCACATTCACCACCATCTC
*GAPDH*	/	F: GAACGGGAAGCTCACTGG R: GCCTGCTTCACCACCTTCT
*mmu‐let‐7e‐5p*	GTCGTATCCAGTGCAGGGTCCGAGGTATTCGCACTGGATACGACAACTAT	F: GCCGGGTGAGGTAGGAGGTTGTATA R: CAGTGCAGGGTCCGAGGTAT
*hsa‐let‐7e‐5p*	GTCGTATCCAGTGCAGGGTCCGAGGTATTCGCACTGGATACGACAACTAT	F: GCCGTGAGGTAGGAGGTTG R: CAGTGCAGGGTCCGAGGTAT
*U6*	GTCGTATCCAGTGCAGGGTCCGAGGTATTCGCACTGGATACGACAAAATATGGAAC	F: TGCGGGTGCTCGCTTCGGCAGC R: CAGTGCAGGGTCCGAGGTAT

### Luciferase reporter assay

The sequences of *SNHG12* contain the *let‐7e‐5p* target site, and their corresponding mutations (MUT) were designed, synthesized and then inserted into luciferase reporter vector GP‐miRGLO, named *SNHG12*‐WT and *SNHG12*‐MUT, respectively (RIBOBIO, Guangzhou, China). All these plasmids were cotransfected with *hsa‐let‐7e‐5p* mimics or *hsa‐let‐7e‐5p* mimics negative control (NC). The relative luciferase activity was examined by Dual Luciferase Assay Kit (Promega, USA) according to the manufacturer’s protocol.

### Statistical analysis

graphpad prism 6.0 (GraphPad Software, CA, USA) was performed for the statistical analysis. All data were analyzed using the Kolmogorov‐Smirnov normality test for normality and presented as the mean standard error of mean. Comparisons between two groups were analyzed by Student’s *t*‐test, and multiple comparisons were analyzed by one‐way ANOVA. A *P* value <0.05 was considered significant. Notably, bioinformatics analysis was mostly performed through the earlier‐mentioned online databases, bioinformatics tools and R language.

## Results

### Sample information processing and identification of DEGs

**Fig. 1:**
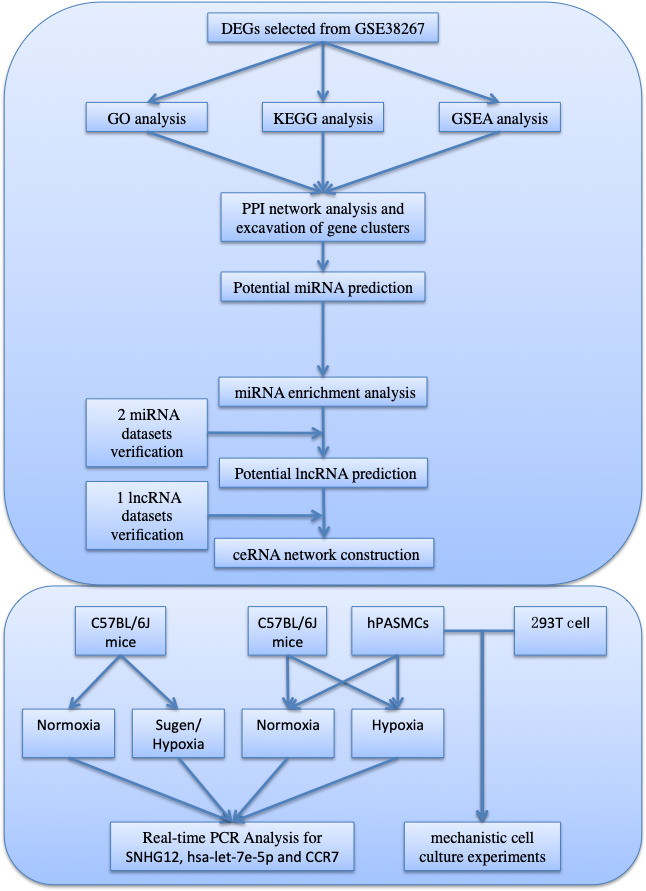
The overall workflow in our study.

**Fig. 2:**
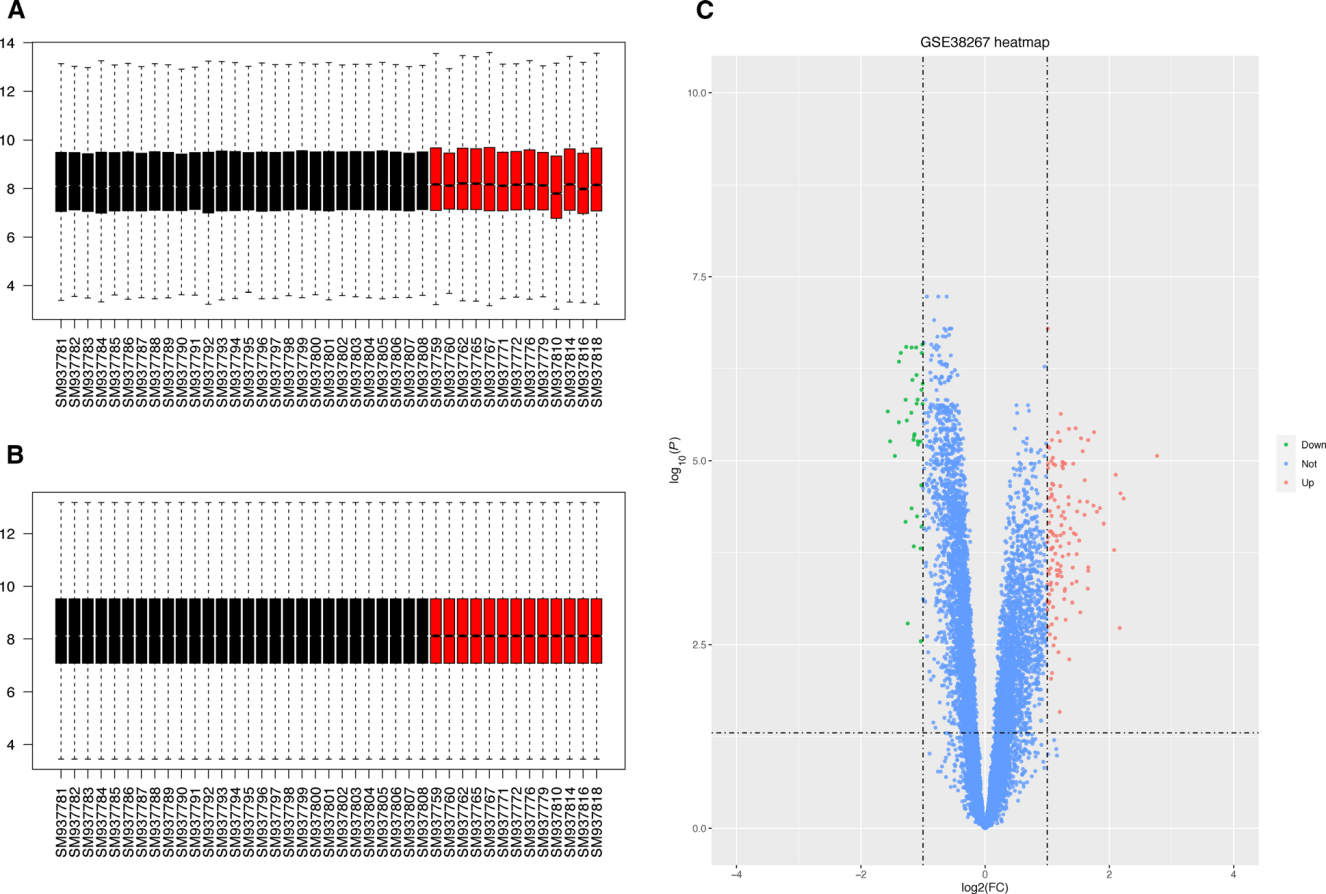
DEGs identification. (A) The boxplot of GSE38267 prestandardization. (B) The boxplot of GSE38267 poststandardization. (C) The volcano plot of DEGs in PAH and HC samples, with green dots representing significantly down‐regulated genes in PAH samples and red dots representing significantly up‐regulated genes in PAH samples.

The general workflow is depicted in a flowchart (Fig. 1). First, the raw data of GSE38267 were downloaded from the GEO database. A total of 41 blood samples were chosen from GSE38267 containing 13 PAH samples and 28 HC samples. The original data were imputed into r software and normalized by the “limma” package (Fig. 2A,B).

According to the data matrix, 165 DEGs were extracted from the samples, among which 129 genes were up‐regulated and 36 genes were down‐regulated (*P* < 0.05, |FC| > 2). Based on the analysis of gene expression, a volcano plot was used to visualize DEGs between two groups (Fig. 2C).

### The DEGs in the PAH samples were mostly enriched in immune‐related response

**Fig. 3:**
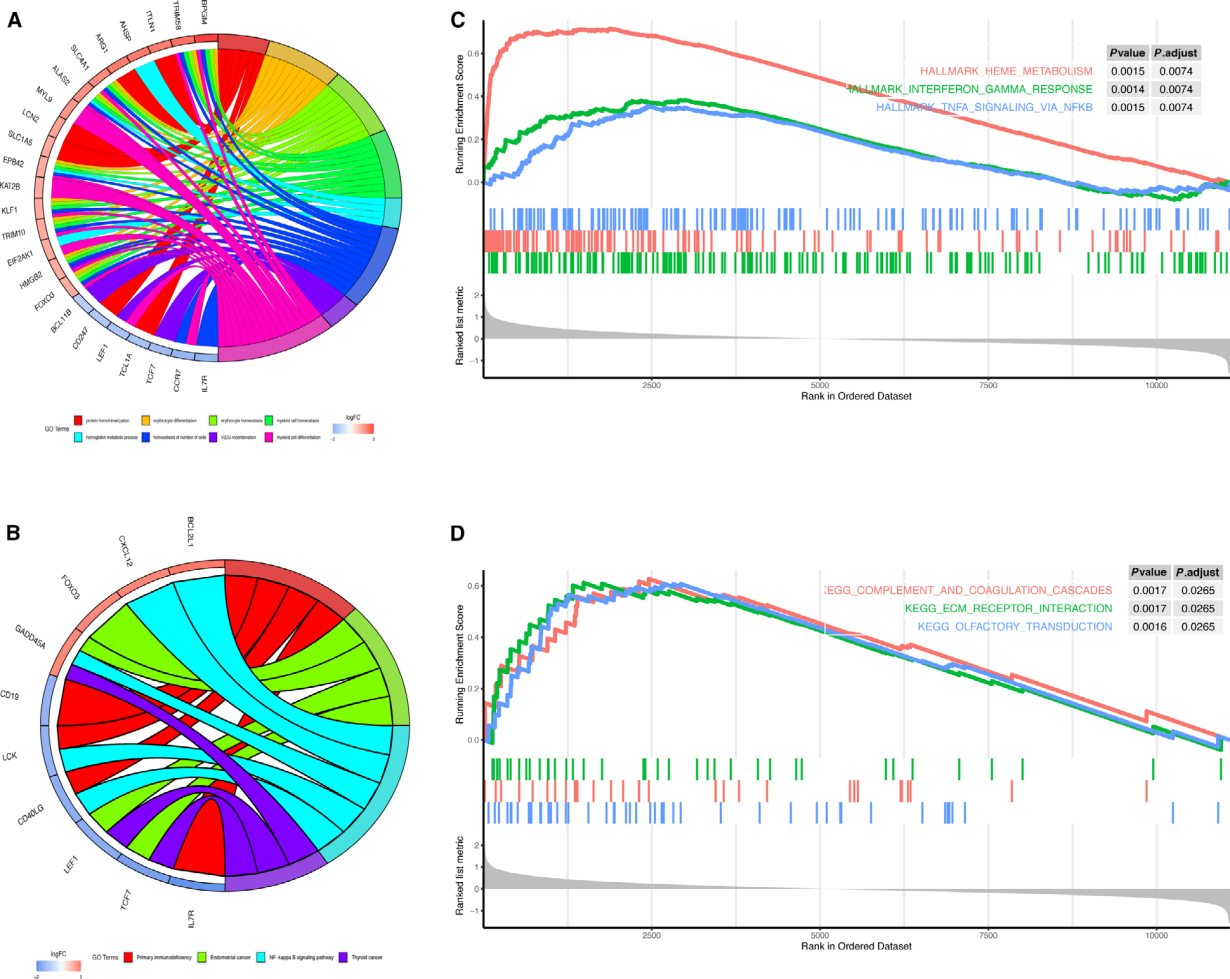
The DEGs in the PAH samples were mostly enriched in immune‐related response. (A) Chord diagrams showed the GO analysis for DEGs representing an association between impacted GO terms and related DEGs. (B) Chord diagrams showed the KEGG analysis for DEGs to investigate the potential pathways. (C) GSEA analysis using h.all.v 6.2.symbols.gmt [Hallmarks] gene set. (D) GSEA analysis using the c2.all.v7.1.symbols.gmt [KEGG] gene set.

To comprehensively explore the potential biological functions and pathways of DEGs, we performed GO and KEGG pathway enrichment analyses, respectively. GO enrichment analysis of DEGs revealed that these genes were mainly involved in myeloid cell differentiation, homeostasis of number of cells and erythrocyte homeostasis (*P* < 0.05) (Fig. 3A). As to KEGG, the nuclear factor‐κB (NF‐κB) signaling pathway and primary immunodeficiency were mostly associated with the DEGs (Fig. 3B). To further validate our results, we performed GSEA. As shown in Fig. 3C, GSEA on the hallmark gene set proclaimed that the genes were mainly enriched in heme metabolism, interferon γ response and TNF‐α signaling via NF‐κB. As for GSEA using the KEGG gene set, the result showed high enrichment of complement and coagulation cascades, extracellular matrix receptor interaction and olfactory transduction (Fig. 3D). Taken together, the results showed that inflammation might play an important role in the occurrence and development of PAH.

### PPI network construction and excavation of gene clusters

**Fig. 4:**
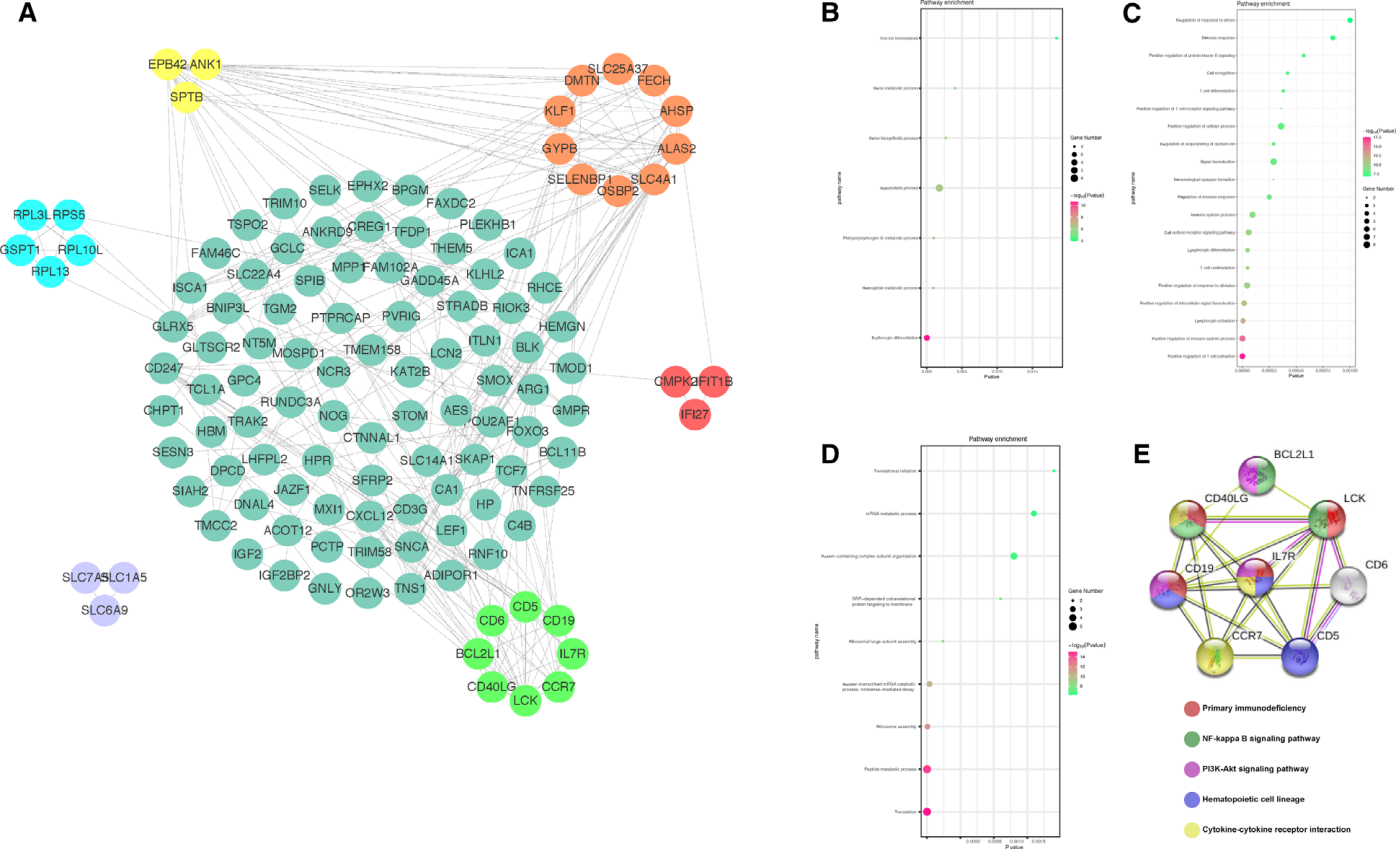
PPI network construction and gene cluster enrichment analyses. (A) PPI network was performed with STRING, and different gene clusters were processed by MCODE plug‐in with cytoscape v.3.7.1. (B–D) Biological process analysis of genes in cluster 1 (B), cluster 2 (C) and cluster 3 (D). The closer the point was to the left, the smaller the *P* value obtained. (E) KEGG pathway analysis of genes in cluster 2. Different colors represented different signaling pathways.

To pick out the core genes, we uploaded 167 DEGs to the STRING for PPI network analysis. Then the data were treated with cytoscape, and gene clusters were identified using MCODE (Fig. 4A and Table 2). It was found that genes in cluster 1 with the highest score were primarily enriched in erythrocyte differentiation, hemoglobin metabolic process and protoporphyrinogen IX metabolic process within the biological process (Fig. 4B). Genes in cluster 2 were mainly involved in positive regulation of T cell activation, positive regulation of immune system process and lymphocyte activation (Fig. 4C). Biological process enrichment analysis of genes in cluster 3 showed that genes were significantly enriched in the translation, peptide metabolic process and ribosome assembly (Fig. 4D). In view of the DEGs enrichment analyses results presented earlier that DEGs in the PAH samples were mostly enriched in immune‐related response, genes in cluster 2 (*BCL2L1*, *CCR7*, *CD5*, *CD6*, *CD19*, *CD40LG*, *IL7R* and *LCK*) related to immune modulation were selected and then verified with KEGG pathway enrichment analysis in STRING. As indicated in Fig. 4E, the result manifested that genes in cluster 2 mainly participated in primary immunodeficiency, hematopoietic cell lineage and NF‐κB signaling pathway. Consequently, a total of eight genes in cluster 2, including *BCL2L1*, *CCR7*, *CD5*, *CD6*, *CD19*, *CD40LG*, *IL7R* and *LCK*, were obtained for further analysis.

**Table 2:** Significant gene clusters were analyzed using MCODE plug‐in. ID, identification number.

Cluster	Score	Nodes	Edges	Node IDs
1	56.0	10	117	*OSBP2, SELENBP1, GYPB, KLF1, DMTN, SLC25A37, FECH, AHSP, ALAS2, SLC4A1*
2	38.0	8	81	*CD40LG, BCL2L1, CD6, CD5, CD19, IL7R, CCR7, LCK*
3	20.0	5	23	*GSPT1, RPL3L, RPS5, RPL10L, RPL13*
4	12.5	3	36	*EPB42, ANK1, SPTB*
5	6.0	3	8	*IFIT1B, IFI27, CMPK2*
6	6.0	3	6	*SLC6A9, SLC7A5, SLC1A5*

### Potential miRNA mining and enrichment analysis

**Fig. 5:**
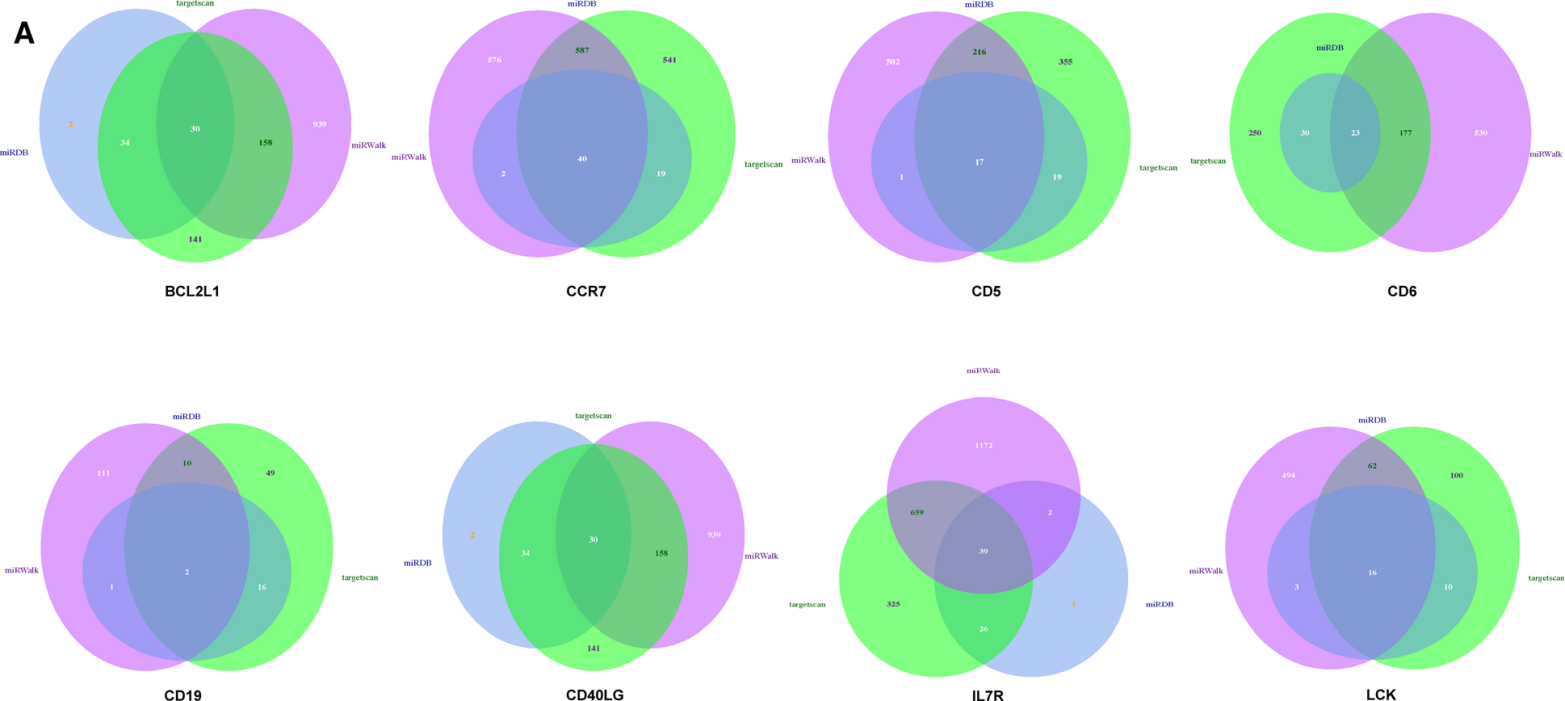
Predictive miRNA. Venn diagram of the predicted miRNA for each mRNA (*BCL2L1, CCR7, CD5, CD6, CD19, CD40LG, IL7R, LCK*) using three online databases (miRWalk, miRDB and TargetScan).

**Fig. 6:**
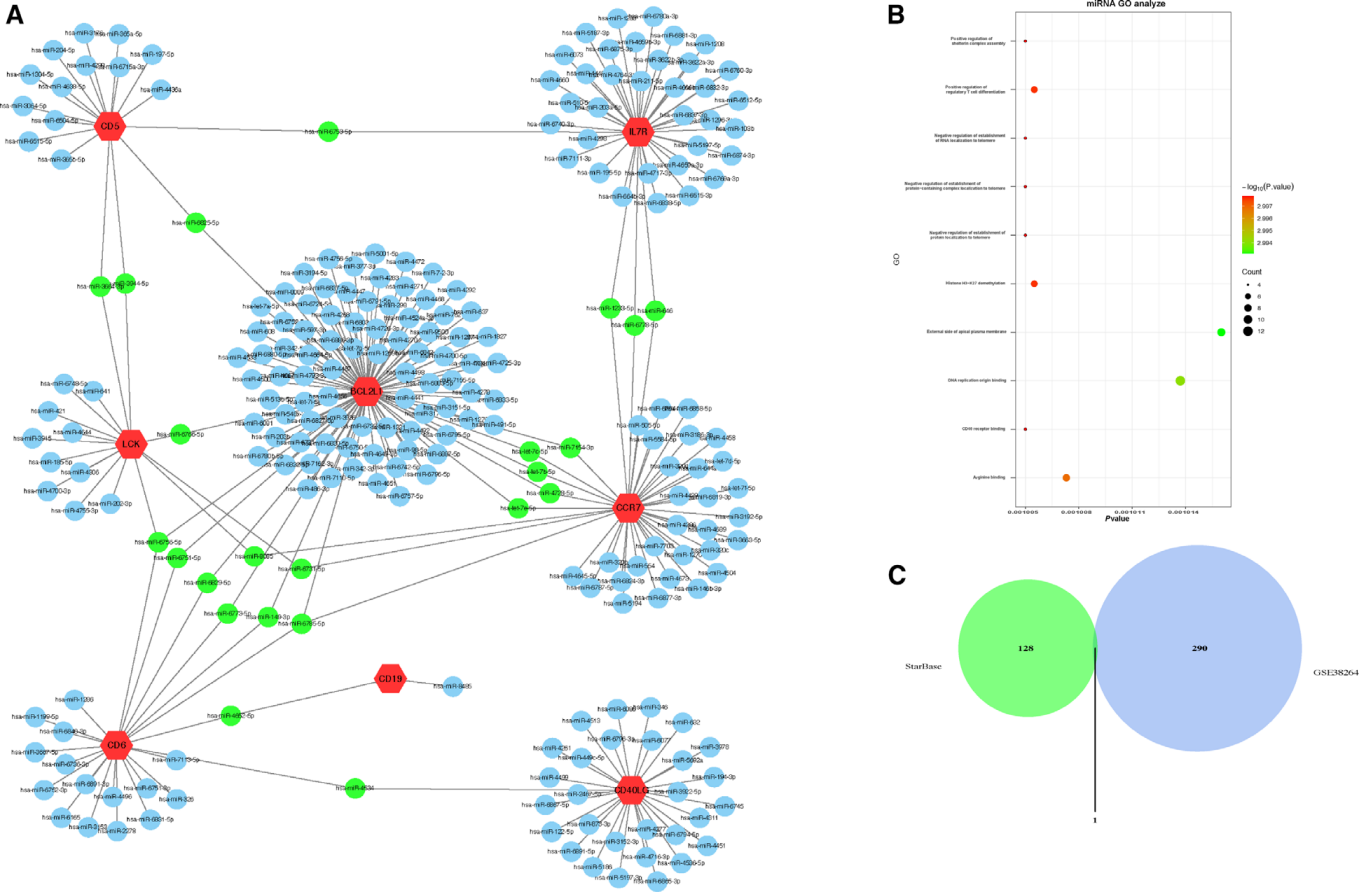
miRNAs prediction, miRNAs enrichment analysis and lncRNAs prediction. (A) Interaction network between eight genes in cluster 2 and its targeted miRNAs. Genes were colored in red; miRNAs targeting only one gene were colored in blue; 23 miRNAs targeting more than two genes were colored in green. (B) Biological process analysis of 23 miRNAs. The closer the point was to the left, the smaller the *P* value obtained. (C) The intersection of the 291 differentially expressed lncRNAs selected from GSE38267 and 129 lncRNAs predicted by starBase.

Based on previous work, eight genes in cluster 2 were respectively uploaded into miRWalk, miRDB and TargetScan, and the intersection of miRNA forecasted by three online tools was chosen as the prediction result (Fig. 5). In addition, the interaction network of a total of 263 miRNAs was reconstructed by cytoscape v3.7.1, and 23 miRNAs were identified with a high number of gene cross‐links (≥2) (Fig. 6A and Table 3). To further evaluate the potential enrichment pathways of candidate miRNAs, we performed miEAA 2.0 with 23 miRNAs. The result showed that miRNAs were mainly enriched in the inflammation‐related terms, such as CD40 receptor binding (Fig. 6B).

**Table 3:** miRNAs and their direct target genes.

miRNA	Genes targeted by miRNA	Gene count
*hsa‐miR‐6753‐5p*	*CD5, IL7R*	2
*hsa‐miR‐6825‐5p*	*CD5, BCL2L1*	2
*hsa‐miR‐3664‐3p*	*CD5, LCK*	2
*hsa‐miR‐3944‐5p*	*CD5, LCK*	2
*hsa‐miR‐6766‐5p*	*BCL2L1, LCK*	2
*hsa‐miR‐6756‐5p*	*BCL2L1, LCK, CD6*	3
*hsa‐miR‐6731‐5p*	*LCK, CCR7*	2
*hsa‐miR‐8085*	*LCK, CCR7*	2
*hsa‐miR‐4652‐5p*	*CD19, CD6*	2
*hsa‐miR‐149‐3p*	*CD6, BCL2L1*	2
*hsa‐miR‐6751‐5p*	*CD6, BCL2L1*	2
*hsa‐miR‐6829‐5p*	*CD6, BCL2L1*	2
*hsa‐miR‐6773‐5p*	*CD6, BCL2L1*	2
*hsa‐miR‐4534*	*CD40LG, CD6*	2
*hsa‐miR‐6785‐5p*	*CD6, CCR7, BCL2L1*	3
*hsa‐miR‐4728‐5p*	*BCL2L1, CCR7*	2
*hsa‐let‐7c‐5p*	*BCL2L1, CCR7*	2
*hsa‐let‐7e‐5p*	*BCL2L1, CCR7*	2
*hsa‐miR‐7154‐3p*	*BCL2L1, CCR7*	2
*hsa‐let‐7b‐5p*	*BCL2L1, CCR7*	2
*hsa‐miR‐6778‐5p*	*CCR7, IL7R*	2
*hsa‐miR‐646*	*CCR7, IL7R*	2
*hsa‐miR‐1233‐5p*	*CCR7, IL7R*	2

In addition, for further screening of meaningful miRNA, raw data of GSE44145 and GSE67597 were downloaded from the GEO database, and DEGs were identified using r software (*P* < 0.05, |FC| >1). As shown in Table 4, it was found that *hsa‐miR‐149‐3p* and *has‐let‐7e‐5p* were up‐regulated in PAH samples in both the dataset from GSE44145 and the dataset from GSE67597, whereas *hsa‐miR‐646* was down‐regulated in both the dataset from GSE44145 and the dataset from GSE67597. However, expression of other miRNAs had no meaningful difference between control samples and PAH samples, or the expression differences of miRNAs in the two datasets were inconsistent. Therefore, *hsa‐miR‐149‐3p*, *has‐let‐7e‐5p* and *hsa‐miR‐646* had higher reliability.

**Table 4:** Verification of candidate miRNAs through GSE44145 and GSE67597.

miRNA	mRNA	Log(FC) [GSE44145]	Log(FC) [GSE67597]	lncRNA prediction in starBase
*hsa‐miR‐6753‐5p*	*CD5, IL7R*	no significance	no significance	No
*hsa‐miR‐6825‐5p*	*CD5, BCL2L1*	no significance	no significance	No
*hsa‐miR‐3664‐3p*	*CD5, LCK*	no significance	−5.3290E−15	No
*hsa‐miR‐3944‐5p*	*CD5, LCK*	0.3432	−0.8840	No
*hsa‐miR‐6766‐5p*	*BCL2L1, LCK*	no significance	no significance	No
*hsa‐miR‐6756‐5p*	*BCL2L1, LCK, CD6*	no significance	no significance	No
*hsa‐miR‐6731‐5p*	*LCK, CCR7*	no significance	no significance	No
*hsa‐miR‐8085*	*LCK, CCR7*	no significance	no significance	No
*hsa‐miR‐4652‐5p*	*CD19, CD6*	no significance	−0.6861	No
*hsa‐miR‐149‐3p*	*CD6, BCL2L1*	0.1904	0.1252	No
*hsa‐miR‐6751‐5p*	*CD6, BCL2L1*	no significance	no significance	No
*hsa‐miR‐6829‐5p*	*CD6, BCL2L1*	no significance	no significance	No
*hsa‐miR‐6773‐5p*	*CD6, BCL2L1*	no significance	no significance	No
*hsa‐miR‐4534*	*CD40LG, CD6*	−0.2778	0.4174	No
*hsa‐miR‐6785‐5p*	*CD6, CCR7, BCL2L1*	no significance	no significance	No
*hsa‐miR‐4728‐5p*	*BCL2L1, CCR7*	−0.0219	1.8762	No
*hsa‐let‐7c‐5p*	*BCL2L1, CCR7*	no significance	no significance	Yes
*hsa‐let‐7e‐5p*	*BCL2L1, CCR7*	0.6242	0.0953	Yes
*hsa‐miR‐7154‐3p*	*BCL2L1, CCR7*	no significance	no significance	No
*hsa‐let‐7b‐5p*	*BCL2L1, CCR7*	0.5005	−0.0893	Yes
*hsa‐miR‐6778‐5p*	*CCR7, IL7R*	no significance	no significance	No
*hsa‐miR‐646*	*CCR7, IL7R*	−4.5295	−0.7849	No
*hsa‐miR‐1233‐5p*	*CCR7, IL7R*	no significance	no significance	No

### lncRNA prediction and ceRNA network construction

To explore the corresponding lncRNA of miRNA, we uploaded 23 miRNAs to starBase to predict lncRNAs, whereas only *hsa‐let‐7e‐5p*, *hsa‐let‐7b‐5p* and *hsa‐let‐7c‐5p* can forecast lncRNAs (Table 4). Combined with previous research that *hsa‐miR‐149‐3p*, *has‐let‐7e‐5p* and *hsa‐miR‐646* had relatively high reliability, *hsa‐let‐7e‐5p* was chosen to do further research, and its related 129 lncRNAs were selected with the highest reliability (very high stringency ≥ 5) via starBase. To further identify promising targets of *hsa‐let‐7e‐5p*, we performed differentially expressed lncRNAs from GSE38267. After cross‐linking between 129 lncRNAs predicted by starBase and 291 differentially expressed lncRNAs of GSE38267, *SNHG12* was found to be a more creditable biomarker (Fig. 6C).

**Fig. 7:**
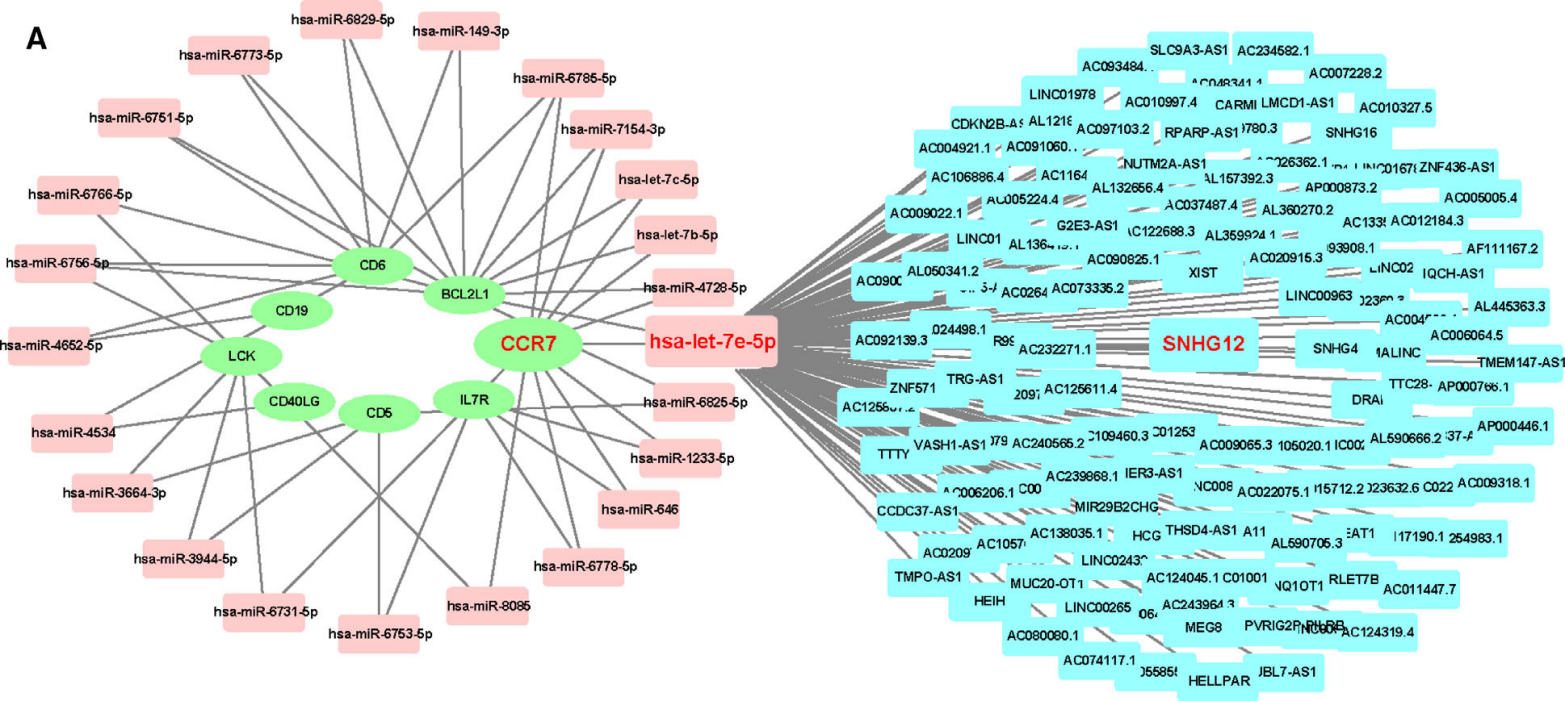
Construction of the ceRNA network and verification of *SNHG12*, *hsa‐let‐7e‐5p* and *CCR7*. (A) The lncRNA–miRNA–mRNA ceRNA network; 8 mRNAs in cluster 2 were labeled green; 23 miRNAs were labeled pink; lncRNAs related to *hsa‐let‐7e‐5p* were labeled blue.

Because miRNA targeting mRNA often results in mRNA degradation, there is a reverse expression relationship between miRNA and mRNA. In our study, the expression of *hsa‐let‐7e‐5p* targeting *BCL2L1* and *CCR7* was up‐regulated in PAH samples. Simultaneously, through the analysis of DEGs from GSE38267, *BCL2L1* was elevated in blood of patients with PAH, whereas the expression of *CCR7* was remarkably reduced in the patients with PAH. Therefore, *CCR7* might act as a reliable potential biomarker for PAH following the successful construction of a ceRNA network (Fig. 7).

### Verification of potential biomarkers expression using qRT‐PCR

**Fig. 8:**
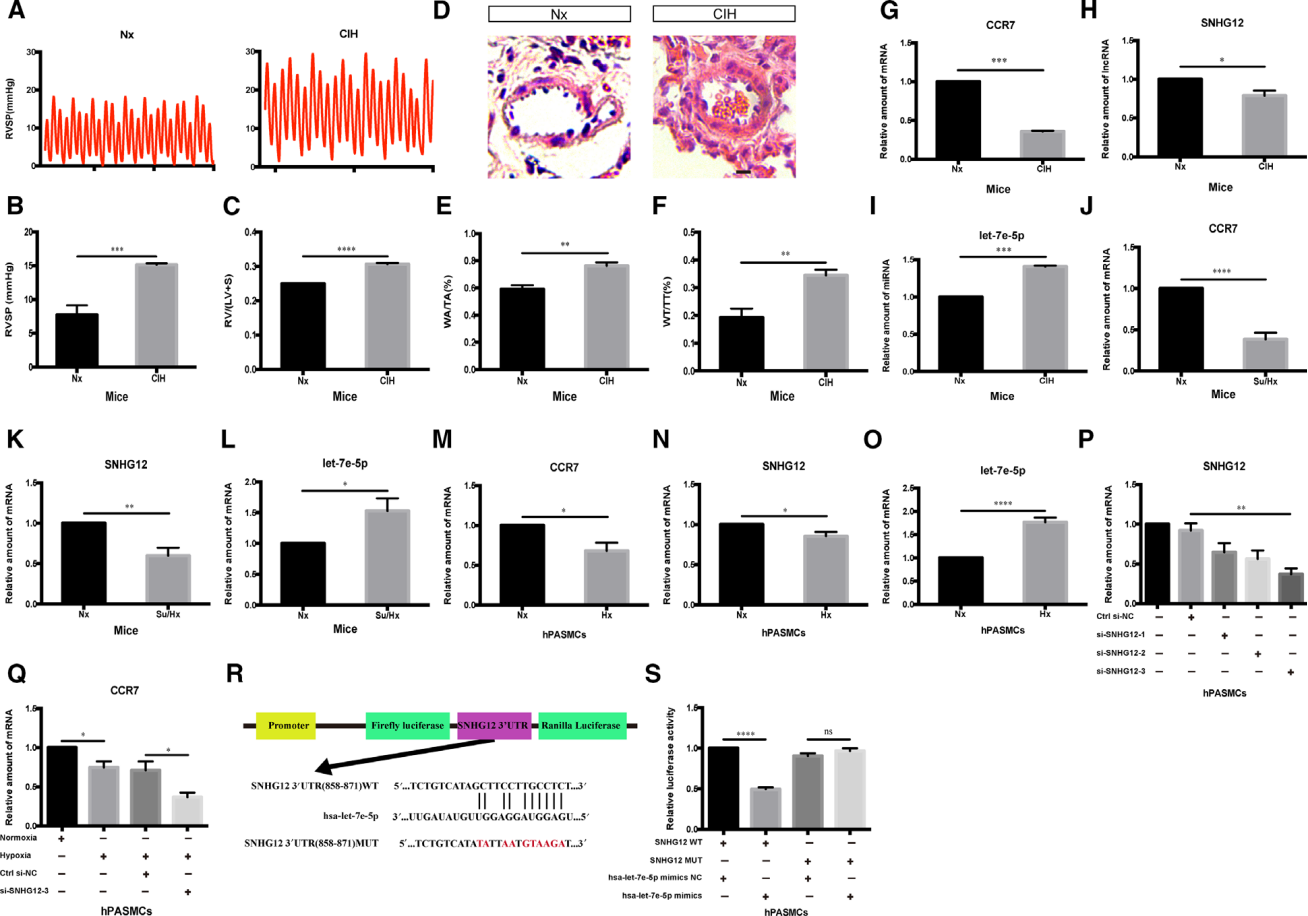
Verification of candidate biomarkers *in vivo* and *in vitro*. (A) Representative images of RVSP in the Nx group and CIH group. RVSP (B; *n* = 6) values and (RV/[LV + S]) (C; *n* = 6) were analyzed for each group. (D) Hematoxylin and eosin staining of pulmonary arteries in lung paraffin sections. Ratio of the pulmonary artery wall area to the total area (WA/TA) values (E; *n* = 6) and ratio of wall thickness to the total thickness (WT/TT) values (F; *n* = 6) were analyzed for each group. (G) qRT‐PCR analysis of *CCR7* expression in the CIH mouse model (*n* = 6). (H) qRT‐PCR analysis of *SNHG12* expression in the CIH mouse model (*n* = 6). (I) qRT‐PCR analysis of *let‐7e‐5p* expression in the CIH mouse model (*n* = 6). (J) qRT‐PCR analysis of *CCR7* expression in the Su/Hx mouse model (*n* = 5). (K) qRT‐PCR analysis of *SNHG12* expression in the Su/Hx mouse model (*n* = 5). (L) qRT‐PCR analysis of *let‐7e‐5p* expression in the Su/Hx mouse model (*n* = 5). (M) qRT‐PCR analysis of *CCR7* expression in hPASMCs (*n* = 5). (N) qRT‐PCR analysis of *SNHG12* expression in hPASMCs (*n* = 5). (O) qRT‐PCR analysis of *let‐7e‐5p* expression in hPASMCs (*n* = 5). (P) Verification of si‐*SNHG12* silencing efficiency in hPASMCs (*n* = 5). (Q) qRT‐PCR analysis of *CCR7* expression after *SNHG12* silence in hPASMCs (*n* = 5). (R) Schematic illustration of *SNHG12*‐WT and *SNHG12*‐MUT luciferase reporter vectors. (S) The relative luciferase activities were detected in hPASMCs after cotransfection with *SNHG12*‐WT or *SNHG12*‐MUT and mimics or NC, respectively (*n* = 5). Scale bars: 20 µm. Comparisons between two groups were analyzed by Student’s *t*‐test, and multiple comparisons were analyzed by one‐way ANOVA. Values are means ± standard error of mean. *P < 0.05; **P < 0.01; ***P < 0.001; ****P < 0.0001.

To verify successful establishment of the CIH‐induced pulmonary hypertension model, we measured the hemodynamics and right ventricular hypertrophy of the Nx group and CIH group. As shown in Fig. 8A,B, RVSP in the CIH group was increased compared with the Nx group. Similarly, the RV/LV + S in the CIH group was notably higher than in the Nx group (Fig. 8C). Moreover, the results of hematoxylin and eosin staining showed that the thickness of the pulmonary artery wall and the degree of muscularization in the CIH group were higher than in the Nx group (Fig. 8D–F). The aforementioned results indicated that the CIH model was successfully established.

To unravel the expression levels of *CCR7*, *hsa‐let‐7e‐5p* and *SNHG12* *in vivo*, we performed qRT‐PCR analysis. As for CIH models, consistent with our prediction, the results showed that there was obvious decline of *CCR7* and *SNHG12* in the CIH group (Fig. 8G,H), whereas the expression of *let‐7e‐5p* significantly increased under intermittent hypoxia treatment (Fig. 8I). Then, we further assessed the expression of *CCR7*, *let‐7e‐5p* and *SNHG12* in the Su/Hx‐induced pulmonary hypertension mouse model. As shown, the expression of *CCR7* and *SNHG12* exhibited a significant decrease in the Su/Hx group concomitant with an increase in *let‐7e‐5p* expression (Fig. 8J–L). To verify our results, we carried out *in vitro* experiments in hPASMCs. As expected, *CCR7* and *SNHG12* were obviously decreased with hypoxia exposure compared with the Nx group in hPASMCs (Fig. 8M,N). Also, there was significant up‐regulation of *let‐7e‐5p* in hPASMCs under hypoxia treatment (Fig. 8O). As such, consistent with previous research, these results offered important insights into the possibility of *SNHG12*, *hsa‐let‐7e‐5p* and *CCR7* acting as potential biomarkers of PAH.

### lncRNA *SNHG12* played a ceRNA role in regulating *CCR7* expression by binding to *hsa‐let‐7e‐5p*

To further explore the interaction among *SNHG12*, *hsa‐let‐7e‐5p* and *CCR7*, we performed a series of experiments. As shown in Fig. 8P, *SNHG12* expression was knocked down by a specific siRNA. Subsequently, the results showed that the knockdown of *SNHG12* reduced the expression of *CCR7* after hypoxia treatment (Fig. 8Q). Then, to confirm the bioinformatics prediction analysis, we applied dual‐luciferase reporter assay in 293T cells. The *SNHG12*‐WT and mutant version without *hsa‐let‐7e‐5p* binding sites were subcloned into GP‐miRGLO plasmids (Fig. 8R). The results indicated the luciferase activity of the *SNHG12*‐WT group obviously decreased in the *hsa‐let‐7e‐5p* mimics group, although there was no difference among *hsa‐let‐7e‐5p* mimics group and NC group in the luciferase activity of the *SNHG12*‐MUT group (Fig. 8S). Rounding up, lncRNA *SNHG12* might function as a molecular sponge in modulating the expression of *hsa‐let‐7e‐5p* to further regulate *CCR7*.

## Discussion

PAH is a lethal pulmonary vascular disease with complex pathogenic mechanisms caused by multiple risk factors. In this study, a total of 41 blood samples from GSE38267 were selected to screen out potential biomarkers in PAH. After a series of bioinformatics analysis, *SNHG12* served as the ceRNA of *CCR7* via sponging *hsa‐let‐7e‐5p*. Meanwhile, *in vivo* and *in vitro* experiments validated the possibility of *CCR7*, *hsa‐let‐7e‐5p* and *SNHG12* as candidate biomarkers for PAH.

Different tissues have different functions and naturally express different RNAs. Therefore, there is a certain difference of RNA expression in blood and tissues. Meanwhile, a firm association and similar expression patterns are recognized between blood and tissues. Gao et al. [11] found that the expression of cysteine‐rich 61 (Cyr61) in the plasma of patients with PAH was highly increased, and similar results were found in lung tissues and PASMCs of the Monocrotaline‐induced PAH rat model. Sun et al. [12] found that the endothelial SCUBE1 expression was decreased by known triggers of PAH, and concentrations of SCUBE1 were also decreased in plasma obtained from PAH rodent models and patients with PAH. Besides, the expression trends of *CCR7* and *SNHG12* were decreased in blood of patients with PAH, lung tissues of PH mouse models and PASMCs under hypoxia exposure, whereas the expression of *let‐7e‐5p* was increased. Hence, in combination with previous studies, some molecules in blood and tissues under pathological conditions may have the similar expression trend, which suggests that these molecules may serve as potential markers for diseases.

As is well known, the main pathogenesis of pulmonary hypertension includes pulmonary vasoconstriction and pulmonary vascular structural reconstruction. In recent years, it is believed that the main pathological changes of pulmonary vascular structural reconstruction include pulmonary vascular endothelial cell (EC) injury, excessive proliferation of smooth muscle cells and fibroblasts, reduction of mitochondrial pathway apoptosis and extracellular matrix proliferation. The inflammatory response inducing the proliferation of the PASMCs caused by the injury of pulmonary vascular ECs may be one of the key factors in pulmonary vascular structural reconstruction. Our data also suggested that the occurrence and development of PAH were correlated with the dysregulation of the immune response pathway. It is well known that NF‐κB is an important transcriptional regulator with various biological activities, which plays a crucial role in immune response, inflammation, cell differentiation and cell proliferation. It has been reported that inflammation of vascular smooth muscle cells played a key role in various vascular disorders, including PAH, restenosis and atherosclerosis [13]. In terms of PAH research, a recent study noted that the up‐regulation of *miR‐335‐3p* mediated by NF‐κB contributes to the inhibition of apelin receptor and development of PAH [14]. Moreover, Li et al. [15] found that NF‐κB‐mediated *miR‐130a* modulation might play a critical role in excessive lung vascular remodeling, which is a major cause of the increased pulmonary vascular resistance. In summary, the inflammation, especially in the NF‐κB pathway, might contribute to the pathogenesis and development of PAH.

Moreover, to validate our results, we constructed PPI network analysis and further excavation of gene clusters to screen out eight genes (*CD40LG*, *BCL2L1*, *CD6*, *CD5*, *CD19*, *IL7R*, *CCR7* and *LCK*) involved in immune system–related function, and *CCR7* was selected as a biomarker candidate. It was reported that *CD40L*, which is also known as *CD40LG*, played an important role in the pathogenesis of PAH through crosstalk between platelets and ECs [16]. The Bcl‐2 family includes antiapoptotic proteins, such as *Bcl‐XL* (*BCL2L1*), and proapoptotic proteins. Chowdhury et al. [17] demonstrated that the deficiency of bone morphogenetic protein receptor type II caused cell‐specific effects, including increasing the expression of *Bcl‐XL* transcripts in PASMCs while inhibiting it in ECs in PAH. There has been little direct evidence that *CD5, CD6* *, CD19* and *IL7R* were involved in the development of PAH, but *CD5, CD6, CD19* and *IL7R* were T cell and B cell surface markers, and the perivascular infiltration of T and B cells correlated with the severity of PAH through aggravating EC damage and pulmonary artery remodeling [18]. Together with our results, it strongly suggests potential roles of these molecules in PAH. LCK, a member of the Src protein kinase family, might play the potential role in PAH induced by protein kinase inhibitors [19]. Also, Larsen et al. [20] found that the *CCR7* deletion in mice deteriorated pulmonary hypertension, including increased RVSP and reduced pulmonary artery acceleration time.

In our research, miRNA *hsa‐let‐7e‐5p* was considered to be a potential biomarker for the diagnosis and treatment of pulmonary hypertension through further verification. *hsa‐let‐7e‐5p* was found to inhibit the progression of head and neck squamous cell carcinoma by significantly decreasing *CCR7* protein expression [21]. Also, the expression of *hsa‐let‐7e‐5p*, targeting FASLG, regulated the function of endothelial progenitor cells in deep vein thrombosis [22]. In addition, accumulating researchers have uncovered the potential value of lncRNAs as biomarkers for disease diagnosis. Based on our current study, lncRNA *SNHG12* was selected as a molecular sponge of *hsa‐let‐7e‐5p* and thus regulates its function. The knockdown of *SNHG12*, highly expressed in the vascular endothelium, could accelerate atherosclerosis [23]. Moreover, Sun et al. [24] revealed that *SNHG12* promoted the proliferation and migration of vascular smooth muscle cells by modulating *miR‐199a‐5p*/HIF‐1α contributing to atherosclerosis. In our study, along with silencing experiments, our dual‐luciferase reporter gene assays indicated that *SNHG12* could directly target *hsa‐let‐7e‐5p*. Taken together, *CCR7*, *hsa‐let‐7e‐5p* and *SNHG12* may be novel biomarkers in PAH.

## Conclusion

Through the research on bioinformatics and experiments *in vivo* and *in vitro*, we detected the pathways and potential molecules related to PAH. Based on the DEGs enrichment analyses, construction of ceRNA and verification of candidate biomarkers, we identified that signal transduction in the immune system played an important role in the occurrence and development of PAH, and *CCR7*, *hsa‐let‐7e‐5p* and *SNHG12* were considered to serve as potential biomarkers in PAH.

## Conflict of interest

The authors declare no conflict of interest.

## Author contributions

MC and XL conceived the idea and wrote the first draft of the manuscript. HD executed the bioinformatics analysis. MC, XL and YW performed the experiments *in vitro* and *in vivo*. MC, XL and XH wrote parts of the manuscript and prepared the tables and figures. XH revised the manuscript. All authors have read and agreed to the published version of the manuscript.

## Data Availability

The data that support the findings of this study are available in GE0 dataset at https:/www.ncbi.nlm.nih.gov/geo/. These data were derived from the following resources available in the public domain: https://www.ncbi.nlm.nih.gov/geo/query/acc.cgi?acc=GSE38267; https://www.ncbi.nlm.nih.gov/geo/query/acc.cgi?acc=GSE44145; https://www.ncbi.nlm.nih.gov/ge/query/acc.cgi?acc=GSE67597.
